# An information theory analysis of spatial decisions in cognitive development

**DOI:** 10.3389/fnins.2015.00014

**Published:** 2015-02-04

**Authors:** Nicole M. Scott, Maria D. Sera, Apostolos P. Georgopoulos

**Affiliations:** ^1^Center for Cognitive Sciences, University of MinnesotaMinneapolis, MN, USA; ^2^Institute of Child Development, University of MinnesotaMinneapolis, MN, USA; ^3^Department of Neuroscience, University of MinnesotaMinneapolis, MN, USA

**Keywords:** information theory, cognitive development, spatial cognition, relational knowledge, language, entropy

## Abstract

Performance in a cognitive task can be considered as the outcome of a decision-making process operating across various knowledge domains or aspects of a single domain. Therefore, an analysis of these decisions in various tasks can shed light on the interplay and integration of these domains (or elements within a single domain) as they are associated with specific task characteristics. In this study, we applied an information theoretic approach to assess quantitatively the gain of knowledge across various elements of the cognitive domain of spatial, relational knowledge, as a function of development. Specifically, we examined changing spatial relational knowledge from ages 5 to 10 years. Our analyses consisted of a two-step process. First, we performed a hierarchical clustering analysis on the decisions made in 16 different tasks of spatial relational knowledge to determine which tasks were performed similarly at each age group as well as to discover how the tasks clustered together. We next used two measures of entropy to capture the gradual emergence of order in the development of relational knowledge. These measures of “cognitive entropy” were defined based on two independent aspects of chunking, namely (1) the number of clusters formed at each age group, and (2) the distribution of tasks across the clusters. We found that both measures of entropy decreased with age in a quadratic fashion and were positively and linearly correlated. The decrease in entropy and, therefore, gain of information during development was accompanied by improved performance. These results document, for the first time, the orderly and progressively structured “chunking” of decisions across the development of spatial relational reasoning and quantify this gain within a formal information-theoretic framework.

## Introduction

Decisions are made across all domains of knowledge, including spatial, perceptual, linguistic, conceptual and social domains. However, these knowledge domains are structured and restructured gradually throughout cognitive development. Piaget was the first to suggest that adaptive mechanisms, such as assimilation and accommodation (Piaget, 1964, 1977), guide the emergence of different cognitive abilities across domains. He suggested that these mechanisms work through the addition and alteration of knowledge structures, and that the subsequent cognitive abilities emerge in a predictable order. However, Piaget was not able to precisely define these mechanisms, yet they remain important for understanding how children eventually come to represent the world. Many researchers have provided evidence in support of Piaget's (1955) stages (Pascual-Leone, 1970; Flavell, 1971; Fischer, 1980; Demetriou et al., 2013), but operationalizing the mechanisms that drive cognitive change through restructuring has remained elusive.

Previous attempts to operationalize these mechanisms were grounded in dynamic systems approaches. For example, Van Geert (1998) used a dynamic systems approach to model Piaget's mechanisms of cognitive change, an approach that describes developmental order as being driven by self-organization (e.g., Thelen and Smith, 1994; Spencer et al., 2012). Stephen et al. (2009) also relied on self-organization as the process driving structural change in their study of cognitive development, and like our approach, they used the construct from information theory known as entropy. Entropy is a powerful approach for studying development, because it provides a method for quantifying how characteristics (e.g., skills, knowledge) are related and change over time.

Information theory was first used to study how information is processed and stored, especially in terms of efficiency (Shannon, 1948; Newell et al., 1958). It has often been applied to the study of memory (Miller, 1956; Simon, 1974; Ericsson et al., 1980; Cowan, 2001), but in this paper we apply information theory—and specifically entropy—to quantify the structural change in developing cognition and to discover the developmental order of those constructs. Specifically, we offer a method for applying it to the emergence of spatial relational reasoning in 5–10 year olds: that is, children's ability to recognize the relative position of one object with respect to another as being *above, below, right*, or *left*.

We chose to investigate spatial relational reasoning because this ability, and spatial thinking more broadly, has been implicated in promoting other cognitive skills (National Research Council, 2006; Uttal et al., 2013a,b). For example, the National Research Council (2006) reported that spatial thinking provided a means of representing a problem abstractly which could then be reasoned about and solved through mental manipulation: a skill that is necessary for problem solving in science and mathematics. In addition, relational reasoning, broadly defined, has been proposed as a cognitive feat that separates humans from other animals, especially when combined with linguistic abilities (Gentner, 2003, 2010). There have been many recent attempts to explain how relative spatial positions are categorized (in infants: Quinn, 1994; Gava et al., 2009; in healthy and disordered cognition: Hayward and Tarr, 1995; Landau and Hoffman, 2005; in computational models: Regier and Carlson, 2001; Lipinski et al., 2012) and how the ability to represent relative location interacts with language (Loewenstein and Gentner, 2005; Dessalegn and Landau, 2008; Ratliff and Newcombe, 2008; Shusterman et al., 2011). Because much previous work has centered on the role of human language in relational reasoning, we investigated the development of spatial relational knowledge across the verbal and nonverbal modalities. Furthermore, we chose to compare *above/below* with *right/left* since these represent spatial relational planes (i.e., vertical and horizontal, respectively) which are learned at different ages. That is, 5 year olds have been shown to know the verbal terms *above* and *below*, but it is typically around 6 or 7 years of age that children master the terms *right* and *left* (Clark, 1980; Cox and Richardson, 1985; Martin and Sera, 2006).

Since spatial relational reasoning is poorly understood in terms of which knowledge domains are in place first (e.g., verbal or nonverbal), and how this knowledge is restructured across development, we were interested in identifying the organizing principles that governed the reorganization of children's knowledge structures. There are a number of different ways in which spatial reasoning could be structured. For example, the knowledge structure of different spatial relations could be based on the specific identity of a specific relation (e.g., above) such that all instantiations of that relation are performed equally well. On the other hand, the modality in which the relation is experienced (verbal or nonverbal), or the plane to which the relation belongs (e.g., vertical or horizontal), could be major factors in organizing spatial relational knowledge. To investigate the nature of changing conceptual organization over 6 years of development, we focused on the information theoretic process known as “chunking.”

Chunking consists of combining individual items into units (chunks) which can then be processed efficiently given their smaller number (Miller, 1956). Miller gave the example of recalling 5 monosyllabic words (e.g., cat, dog, bee, rat, cow) rather than 15 phonemes (or letters: c, a, t, d, o, g, etc.), where words are chunks of 3 phonemes (or letters). Different types of information (e.g., words, images, etc.) can be chunked differently (e.g., as sentences, scenes, etc.) and within different capacities of memory (remembering lists of letters vs. lists of digits; Miller, 1956; Simon, 1974), and the chunking of information is based largely on the current structure of a cognitive hierarchical organization (e.g., Larkin et al., 1980). For example, experts have already organized large chunks of like information together, making for quick retrieval, whereas novices are still learning how to organize information in the new context (e.g., chess: Chase and Simon, 1973). Novices must discover on their own the optimal organization of new information with respect to already held concepts, whether to integrate new concepts with old ones or form new chunks in memory. For example, a chess master “sees” the relationships between pieces on the board (e.g., attack or defend) and combines the position of pieces into meaningful chunks, whereas novices are more likely to remember only the position of single pieces on the board (Chase and Simon, 1973). This suggests that chunking begins on an item-by-item basis with each individual piece of information being processed separately; then, after a set of similar items (or, in the case of chess, positions) have been chunked together, new but similar information can be processed as if it belonged to the previously formed group. Taken together, chunking expedites learning of similar tasks and aids in organizing new knowledge with other knowledge like it. In this sense, chunking makes stored information more accessible and optimizes decision-making. By studying how information is processed and stored over human development, it would be possible to identify which parts of knowledge domains (or specific skills) are acquired first and provide structure to other areas, and at what points in development certain skills are performed similarly and when learning accelerates.

It has been previously demonstrated that infants have some capacity to chunk incoming information according to spatial, perceptual, linguistic, conceptual or social similarities (Feigenson and Halberda, 2008; Stahl and Feigenson, 2014). For example, Feigenson and colleagues (Feigenson and Halberda, 2008; Stahl and Feigenson, 2014) presented 16-month-old infants with arrays of 4–6 same or similar objects (e.g., balls, dolls, cars, or cats) and recorded their looking time as the objects were retrieved, one at a time, after being hidden for a short period. When the objects were spatially or categorically grouped, infants would look longer at the hiding spot for objects that had not yet been retrieved (to come into view), indicating that infants expected at least one more object to be retrieved. Clearly, then, chunking is available to infants; therefore, it is a potentially powerful tool for discovering the order, or progression, of concept development (e.g., in the spatial, perceptual, linguistic, conceptual or social domains). To our knowledge, however, this approach has not been applied to any domain of conceptual development.

In summary, the main goal of this paper is to offer an example of this new approach to the study of cognitive development. We demonstrate the approach using data from one of our current studies on the development of spatial relational knowledge in 5–10 year olds. Specifically, we document how knowledge of the spatial relations *above, below, right*, and *left* changes over development and how these concepts are reorganized with increasing age, knowledge and skill. We used hierarchical clustering analyses to identify conceptual “chunks,” and information theoretic methods to quantify the amount of organization in the cognitive system by measuring the amount of entropy at each age group. Specifically, we document how knowledge of the spatial relations *above, below, right*, and *left* becomes more unified across 6 years of life as different instantiations of these relations gradually become “chunked” together.

We propose that chunking together different instantiations of spatial relational information is at the core of this conceptual change. However, we had no a priori expectation of which aspects of spatial relational information would be chunked together at each age group. Thus, we sought to discover the organizing principles through which chunking acted. As mentioned earlier, there were a number of different organizing principles that could direct chunking (see Methods). We also examined the “chunks” with respect to the amount of entropy, or uncertainty, in the system. Fewer chunks indicate well-structured organization in the system, whereas many chunks indicate little structural organization, and, suggest higher entropy. Our two measures of entropy were: (1) cluster entropy, which involves the number of clusters at each age, and (2) task entropy, which involves the distribution of the 16 tasks across the clusters at each age group. So, the amount of entropy *in the chunks* (i.e., task entropy) could be used as a measure of how the concepts are structured in the cognitive system (i.e., what organizing principles govern each structure), while the entropy *within an age group* (i.e., cluster entropy) reflects the changing amount of structure in the cognitive system with development. Comparing across age groups, we were able to capture the change in structure and gain of information in the cognitive system as development progressed. We hypothesized that chunking would lead to a systematic decrease of the entropy in the cognitive organization of relational concepts as an increasing number of different relational tasks were performed more similarly with increasing age. We expected concomitant improvement in performance across multiple relational tasks with age, culminating with minimum cluster entropy at age 10 years and near adult levels of performance. Remarkably, until our study, verbal and nonverbal knowledge of these four spatial, relational concepts had not been examined in a single cross-sectional study.

## Methods

### Participants

Children between the ages of 5,0 and 10,11 were recruited from the Minneapolis-St. Paul metro area, mostly from middle to high SES Caucasian families. We chose this age range because at the age of 5 years, children have been shown to have some spatial relational knowledge, and at 10 years their relational knowledge should be close to adult levels. Each of 6 age groups (5, 6, 7, 8, 9, 10) included 10 boys and 10 girls for a total of 120 children. To ensure that the entire range within each age group was represented, both younger and older children at each age group participated so that the mean age was at the midpoint for each group. The children had no known cognitive or language deficits. An additional 19 children were tested but their data were not used: 13 participated in pilot work while the 6 other children were discovered to have a known cognitive disorder when they came to the lab or were consistently exposed to a language other than English at home. This research complied with University of Minnesota IRB approval and HIPAA protocols.

### Tasks

Children performed a total of 16 tasks of spatial relational reasoning. Four spatial relations were investigated: *above, below, left*, and *right.* Knowledge of each relation was tested in two conditions within each of two modalities: verbal production, verbal comprehension, nonverbal congruency, and nonverbal incongruency (4 relations × 2 conditions × 2 modalities = 16 tasks; see below for task details). Children always performed the nonverbal tasks before the verbal tasks to avoid priming children with a verbal strategy for the nonverbal tasks and always performed the comprehension tasks last in order to avoid giving children the correct label for each relation before they performed the verbal production tasks. Some may argue that each instantiation of a spatial relation should not be considered separate tasks, but we chose this approach because we were interested in trying to capture how decisions across various instantiations of relational tasks were chunked together as a function of development. Again, we had no a priori expectation of which aspects of spatial relational information (e.g., relation type, relational plane, modality of task, task type, or no pattern) would be chunked together at each age group and from which we could interpret an underlying cognitive mechanism. However, it should be pointed out that this study was focused only on spatial tasks, and, hence, a possible extension of this approach to other domains will need to be explored in future studies.

### Nonverbal conditions

**Figure 1:**
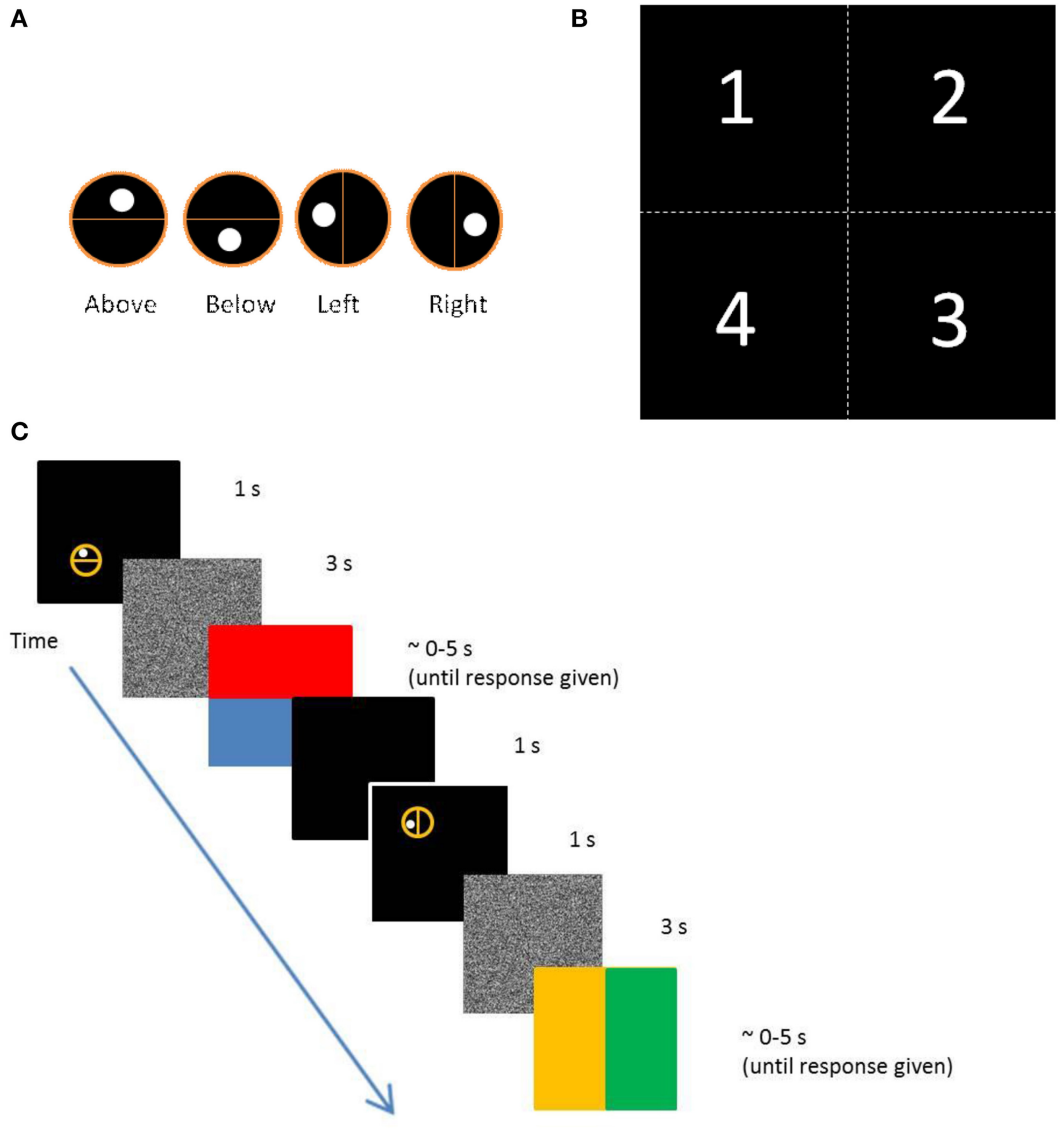
**(A)** Set of all four stimuli types illustrating spatial relations of *above*, *below*, *left*, and *right*. **(B)** The four quadrants in which the stimuli appeared. Every relation appeared in each quadrant three times. Each quadrant was divided into sub-quadrants for a total of 16 possible locations on the computer screen; however, stimuli only appeared in the 12 locations along the perimeter of the screen so stimuli never appeared near the center of the screen. *Above* trials were congruent when the stimulus appeared in quadrants 1 or 2, but incongruent when they appeared in quadrants 3 or 4. The opposite was true for *below* trials. Similarly, trials in which the stimulus for *right* were congruent were when they appeared in quadrants 2 or 3, and incongruent when they appeared in quadrants 1 or 4. The opposite was true for *left* trials. **(C)** Illustration of the screen progression of two trials for the nonverbal task and the amount of time each screen was displayed. The upper-most panel shows the first screen (an example of *above* incongruent trial), the middle panel is the distracter screen (static snow), and the lower-most panel shows the response screen. The second example trial depicts a congruent *left* trial.

We created a computer game which tested children's ability to make nonverbal spatial relational judgments. In these eight tasks, the child had to encode the position of a dot relative to a line and respond by touching a computer screen in the matching relative location. The dot could appear *above*, *below*, *to the left*, or *to the right* of the line (Figure 1A). The dot and line were encompassed by a circle and the whole stimulus could appear in 1 of 4 quadrants on the screen (Figure 1B), but the relative position of the stimulus on the computer screen could either match or contradict the internal relation represented. Therefore, each relation was either congruent or incongruent. Trials where the relative location of the stimulus on the screen matched the relation depicted by the dot and line were congruent (stimulus at top of screen, dot *above* line); trials where these relationships did not match were incongruent (stimulus at top of screen, dot *below* line; see Figure 1C for an example). The child had to remember the dot's relative position for 3 s before responding by touching the computer screen on the same relative side of a new line.

With this design, there were two task conditions that were presented in an intermixed, random order. The first task condition consisted of congruent trials in which simple perceptual matching could be used to correctly respond. The child responded by touching the screen, and in this task they would be correct even if their strategy was to touch exactly where they remember seeing the stimulus, thereby (perhaps) not encoding the relation. The other task condition was the incongruent trials task in which children could only answer correctly by using flexible relational coding. Feedback was provided to children after each trial through audio clips of applause for correct responses and a zapping sound for incorrect trials. Before testing began, children were trained with 8 trials. All children started with the same set of eight training trials before moving on to the test trials. To avoid explicit priming, the experimenter only used the terms “here” and “there” and never said the words *above*, *below*, *right*, or *left* in providing instructions to play the nonverbal computer game. The exact instructions were:

“I am going to show you how to play this computer game. In this game there is a circle and it is going to appear anywhere on the screen. There is going to be a line that goes through the middle of the circle and there is going to be a dot on one side of this line. You need to remember which side of the line you saw the dot. Then the circle is going to disappear and the screen will turn gray and white [visual static]. Then the screen will split in half and each side will be a different color. You touch the screen on the side of the new line that matches the side that the dot appeared on. Are you ready to try a few with me?”

### Verbal conditions

**Figure 2:**
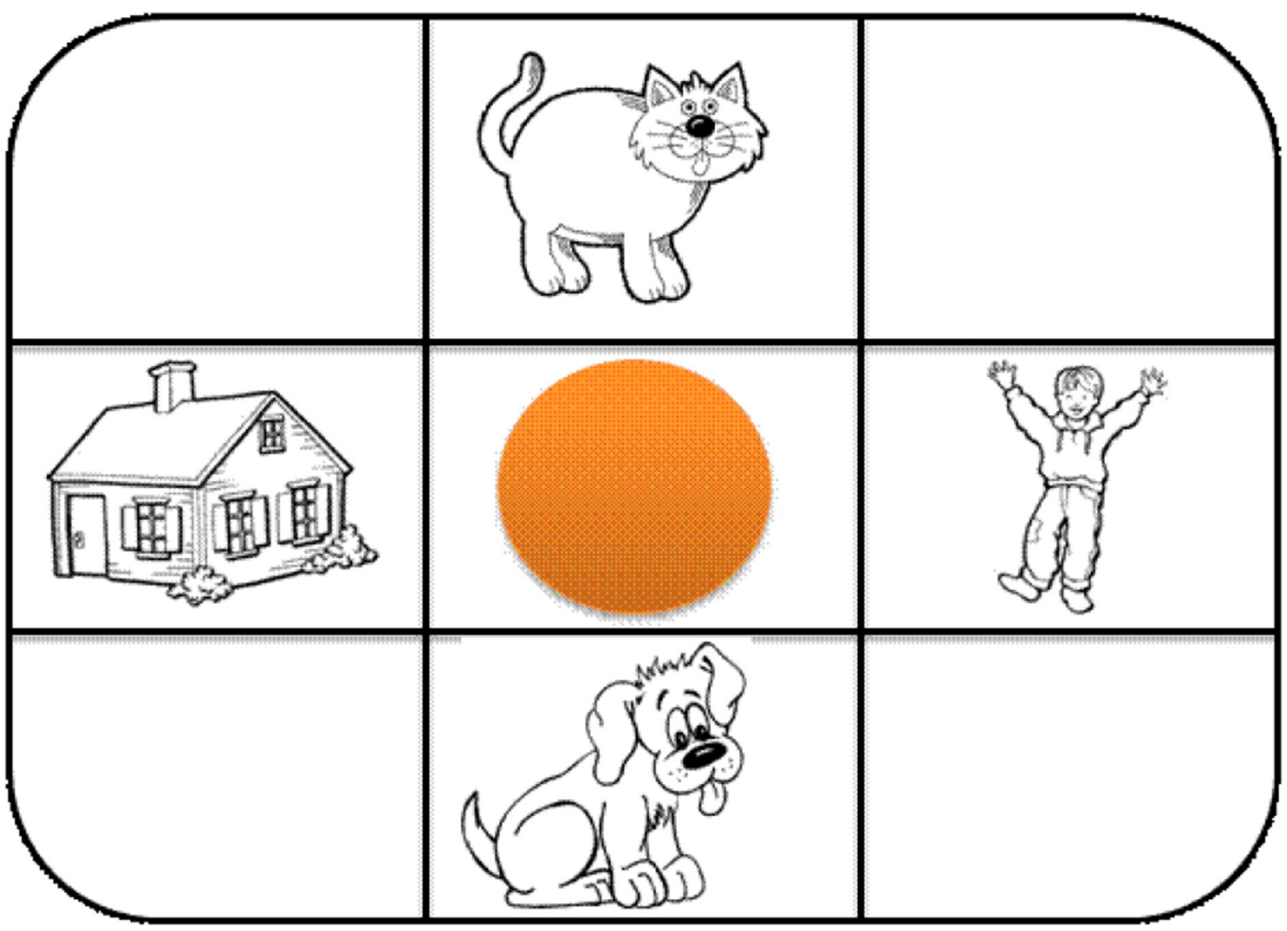
**Illustration of the apparatus for the verbal task**. Each object represents the exact appearance of each magnet. The “cat” is in the *above* position; the “child” is in the *right* position; the “dog” is in the *below* position; and, the “house” is in the *left* position.

The eight verbal tasks consisted of a tic-tac-toe-like magnetic board with a circle in the middle square (modified from Cox and Richardson, 1985). Children were first required to say the location of a magnet on the board with respect to the circle (production), then asked to place a magnet onto the board with respect to the circle (comprehension; see Figure 2). The magnet could be placed *above*, *below*, *to the right*, or *to the left* of the circle. For the production condition, the instructions provided to the child were as follows:

“In this game, I am going to put a magnet on the board and you need to tell [confederate's name or parent] where the magnet is in terms of this circle (experimenter points at the circle). [Confederate's name or parent] cannot see your board so you need to be as specific as possible. If you tell her/him where the magnet is in terms of the circle then s/he will know exactly where to place their magnet so that their board looks just like yours.”

For the comprehension condition, the instructions were as follows: “In this game I am going to tell you where to place a magnet on the board in terms of this circle (experimenter points at the circle). You put the magnet where I tell you in terms of the circle.” Then, children were told, “Put the *boy* (or *dog*, etc.) *above* (or *below, left, right*) the circle.” No feedback was provided to children with respect to correctness of their response. Trials progressed in sets of four wherein each relation was seen only once per set. The order the relations were given within a set was randomized using a Latin square design. Performance in these tasks, and in the nonverbal tasks, was quantified as percent correct.

### Hierarchical tree clustering

We hypothesized that a key mechanism in the development of relational knowledge is the progressive treatment of different tasks as the same (i.e., they become chunked) such that more items should be packed into fewer clusters with increasing age. We tested this hypothesis by conducting a hierarchical tree clustering analysis (Shepard, 1980) of performance on the 16 tasks for each of 6 age groups, separately. We then searched for consistent grouping (i.e., chunking) of task performance to find out whether a knowledge structure had been formed that represented particular organizing principles or task characteristics (e.g., modality, condition, relation or plane). If tasks consistently chunked together from one age to the next (e.g., two tasks chunk at age 5 then appear as a chunk at each subsequent age), then it can be argued that each task that joins the chunk later in development (e.g., age 7) has been assimilated with the previously chunked tasks, or that the chunk at 5 years of age has been modified to accommodate the new skills at the later age.

**Figure 3:**
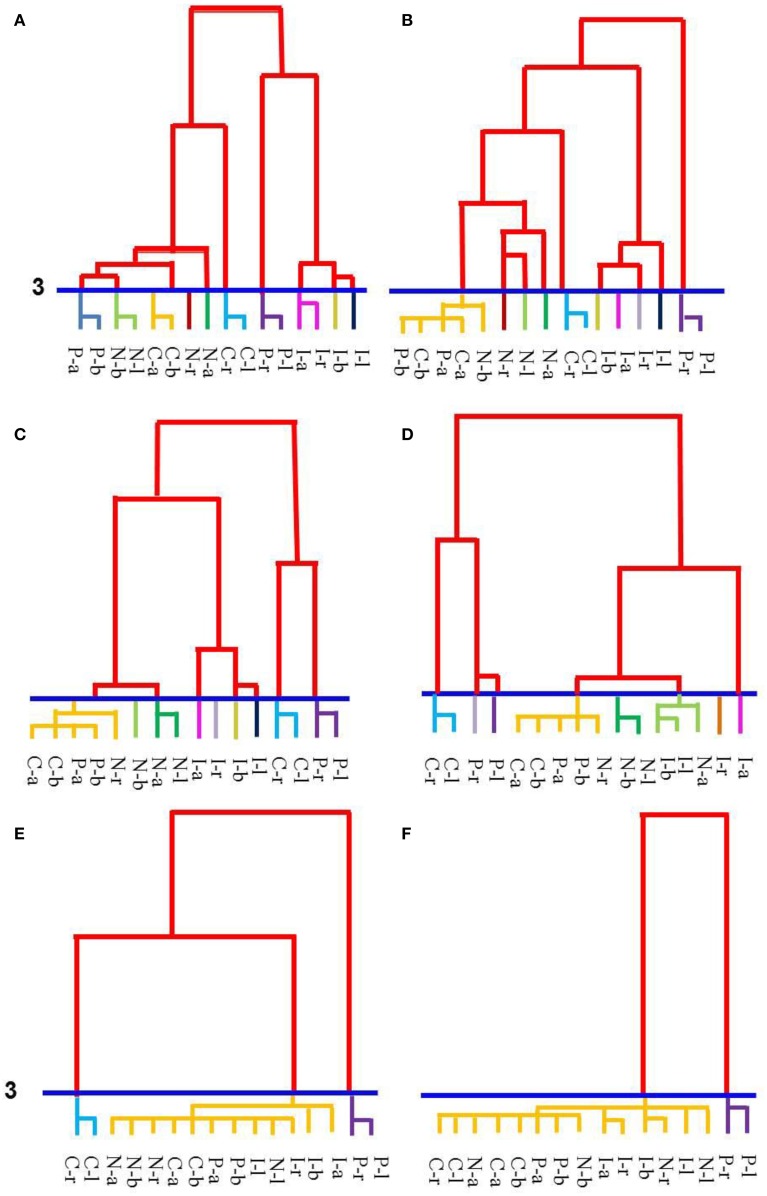
**Hierarchical tree clustering of performances across the 16 tasks for each age group. (A)** The tree for 5-year-olds. **(B)** The tree for 6-year-olds. **(C)** The tree for 7-year-olds. **(D)** The tree for 8-year-olds. **(E)** The tree for 9-year-olds. **(F)** The tree for 10-year-olds. Increasing chunking (i.e., fewer clusters) can be seen as development progresses from age 5 **(A)** to age 10 **(F)**. Clusters were defined as a group of tasks that formed a group below a scale interval of 3 (blue line). Each color at the bottom of the tree demarcates the distinct clusters. Each task is identified by the condition (capital letter) and relation (lower case letter). Key: P, production tasks; C, comprehension tasks; N, congruent trials; I, incongruent trials; a, *above*; b, *below*; r, *right*; l, *left*.

Hierarchical cluster analyses can be used to find the general factors that underlie performance on a set of tasks as it clusters performance according to shared factors and organizes the clusters by their similarity (Shepard, 1988; Corter, 1996). It is a useful analysis for capturing progressive similarity of items. In our study, clusters were formed based on performance as measured by percentage of correct trials. The tasks that fell into a cluster, then, can be judged to be performed similarly well or similarly poorly consistently across all individuals in the group. In hierarchical tree clustering, clusters are scaled as being at a distance from an origin that starts at 0 for items that are the most similar and ends at 25 for items that are the most different. Therefore, the further from the origin that a cluster forms, the less similar the items are within the cluster. Furthermore, items that are placed adjacently along the origin (i.e., at the bottom of each panel in Figure 3) are more closely related than items placed farther apart. To examine the content of the clusters (i.e., tasks) we cut the tree at scale interval 3 (blue line across tree structures in Figure 3) and considered tasks to be chunked only if they formed a cluster by scale interval 2. Below scale interval 3, we considered cluster items (tasks) to be reasonably similar with performance being neither completely equal nor completely different. In this way, items in a cluster could represent tasks performed at 87 and 88% or tasks performed at 45 and 42%, etc. (percentages taken from 5 year olds' dendrogram). Cutting the trees at interval 6 (and counting only clusters formed by scale interval 5)—which makes the items in the clusters less similar to each other—yielded similar results, thus the choice of interval size does not change the results substantially. SPSS for Windows (version 21) was used for this analysis (method: between-group linkage; measure: squared Euclidean distance).

### Cognitive entropy measures

Since relational knowledge is less developed at age 5 years than at later years, we hypothesized that relational concepts would be poorly connected (i.e., not chunked together) at 5 years of age and, therefore, entropy would be highest at that age. We further hypothesized that entropy would decrease with increasing age, and in doing so would capture the structure that is added as knowledge is gained and suggest which different concepts have become connected or unified. Finally, we expected that a decrease in entropy (i.e., reflecting a gain of information) would be accompanied by an improvement in performance.

We identified and measured two sources of entropy. One was *cluster entropy*, *S*_*C*_, referring to the number of clusters in a tree (i.e., at each age group), with respect to the maximum of 16 possible clusters, whereas the other was *task entropy*, *S*_*T*_, referring to the distribution of tasks (*T* = 16) across clusters (at each age group). We calculated each entropy measure separately (Shannon, 1948), assessed their change during development, and evaluated their relation to each other, as follows.

The cluster entropy for a given tree is given by:

$$S_{C}={\text{log}}_{2}N\text{ bits}$$

where *N* is the number of clusters in the tree. For example, for a tree with 16 clusters, *S*_*C*_ = *log*_2_16 = 4 bits, and, for a tree with 2 clusters, *S*_*C*_ = *log*_2_2 = 1 bit. With regard to task entropy, it should be noted that chunking cannot dictate by itself how the chunked items (tasks) would be distributed across the chunks (clusters). For example, if the 16 tasks were distributed across two clusters, then in an isotropic distribution each cluster should contain 8 tasks; however, in any number of different anisotropic distributions the two clusters could contain 10 and 6 tasks or 2 and 14 tasks, etc. For a given tree (age group) with *N* clusters, let *k*_*i*_ be the number of items (tasks) in the *i*th cluster, where *i* = 1, *N*. Then, the task entropy of the *T* = 16 tasks for this tree (in bits), where *p* is the probability of finding that distribution, is:

$$S_{T}=-\sum_{i\;=\;1}^{N}p\left(k_{i}\right)\;{\text{log}}_{2}\;p\left(k_{i}\right)$$

where

$$p\left(k_{i}\right)=\frac{k_{i}}{T}$$

and

$$\text{max }S_{T}=S_{C}$$

Since the maximum task entropy is limited by the number of clusters in a tree, we defined the tree-specific (i.e., age-specific) task entropy *S*′_*T*_ as a fraction of max *S*_*T*_:

$${S^{\prime }}_{T}=\frac{S_{T}}{\text{max }S_{T}}=\frac{S_{T}}{S_{C}}$$

Again, it should be noted that *S*_*C*_ and *S*′_*T*_ are independent of each other (see above). In other words, there is no a priori reason to assume that task entropy should be anything but isotropic (i.e., *S*′_*T*_ = 1). Since *S*′_*T*_ is a fraction, its values range from zero to one.

## Results

First, we discuss the results from the cluster analyses, and the changes that they capture in “chunking” of relational information with development. Then we discuss the changes observed in entropy reduction with development. Within each age group, cluster entropy is discussed first, followed by task entropy.

### Hierarchical clustering

Figure 3 illustrates the results from the hierarchical cluster analyses, with each panel showing the dendrogram observed for each age group. Five-year-olds' performance on the 16 tasks grouped into 10 clusters (Figure 3A). Each set of relational opposites (*above/below* and *right/left*) clustered according to verbal task type (production or comprehension), forming 4 separate groups. The nonverbal tasks of congruent and incongruent formed an additional 2 cluster pairs and 4 single, discrete clusters where each relation formed a cluster within the same task or held a position adjacent to other relations within the same task. For 6-year-olds (Figure 3B), again, 10 distinct clusters emerged. These clusters differed from 5 year olds in two ways. Firstly, the *above/below* verbal tasks collapsed into a single cluster and the nonverbal tasks were performed more variably so each relation formed separate, distinct clusters to form the remaining 7 groups. Few changes appeared between 6 and 7 year olds (Figure 3C), although nonverbal congruent tasks began to merge with the *above/below* verbal tasks cluster, thus reducing the number of clusters to 9 in the 7 year olds' cognitive organization. For 8-year-olds (Figure 3D), all of the verbal tasks aligned closely together along the tree's baseline (horizontal) axis indicating that these were performed more similarly to each other than to the nonverbal tasks, despite still forming 2 separate clusters (i.e., *right/left* comprehension and *above/below* verbal tasks) with production of *right* and *left* splitting as separate items. Similarly, the nonverbal tasks aligned closely along the tree's baseline axis as they began to collapse together, forming an additional 4 clusters (for 8 groups total) according to task condition. For 9 year olds (Figure 3E), only 3 distinct clusters emerged: *right/left* comprehension, *right/left* production, and everything else. Ten year olds (Figure 3F) had only 2 clusters after comprehending *right/left* collapsed with the other tasks, leaving production of *right/left* as a single cluster.

**Figure 4:**
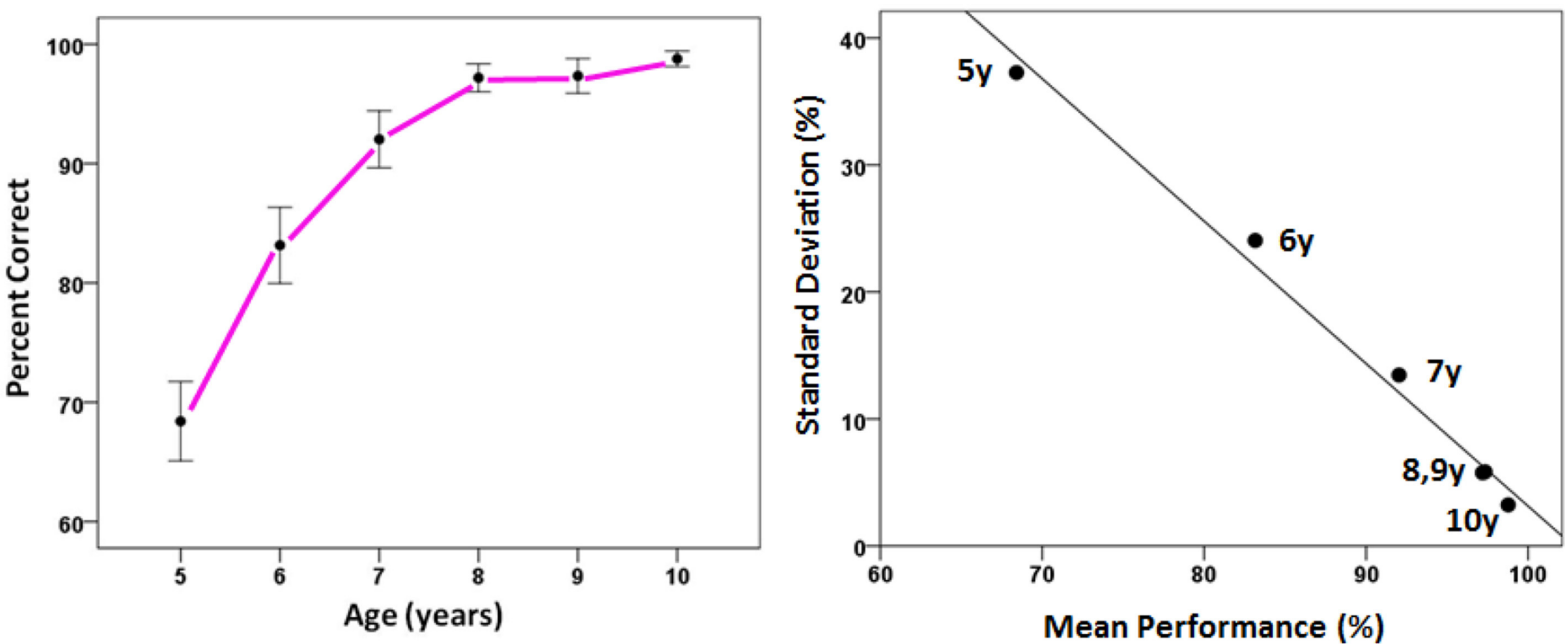
**Performance as a function of age. Left panel:** Correct performance (±SEM) increased with age, while variability decreased (error bars). **Right panel**: Variability in performance decreased with increasing age, and as a linear function of mean correct performance (*r*^2^ = 0.989, *P* = 0.000044).

**Figure 5:**
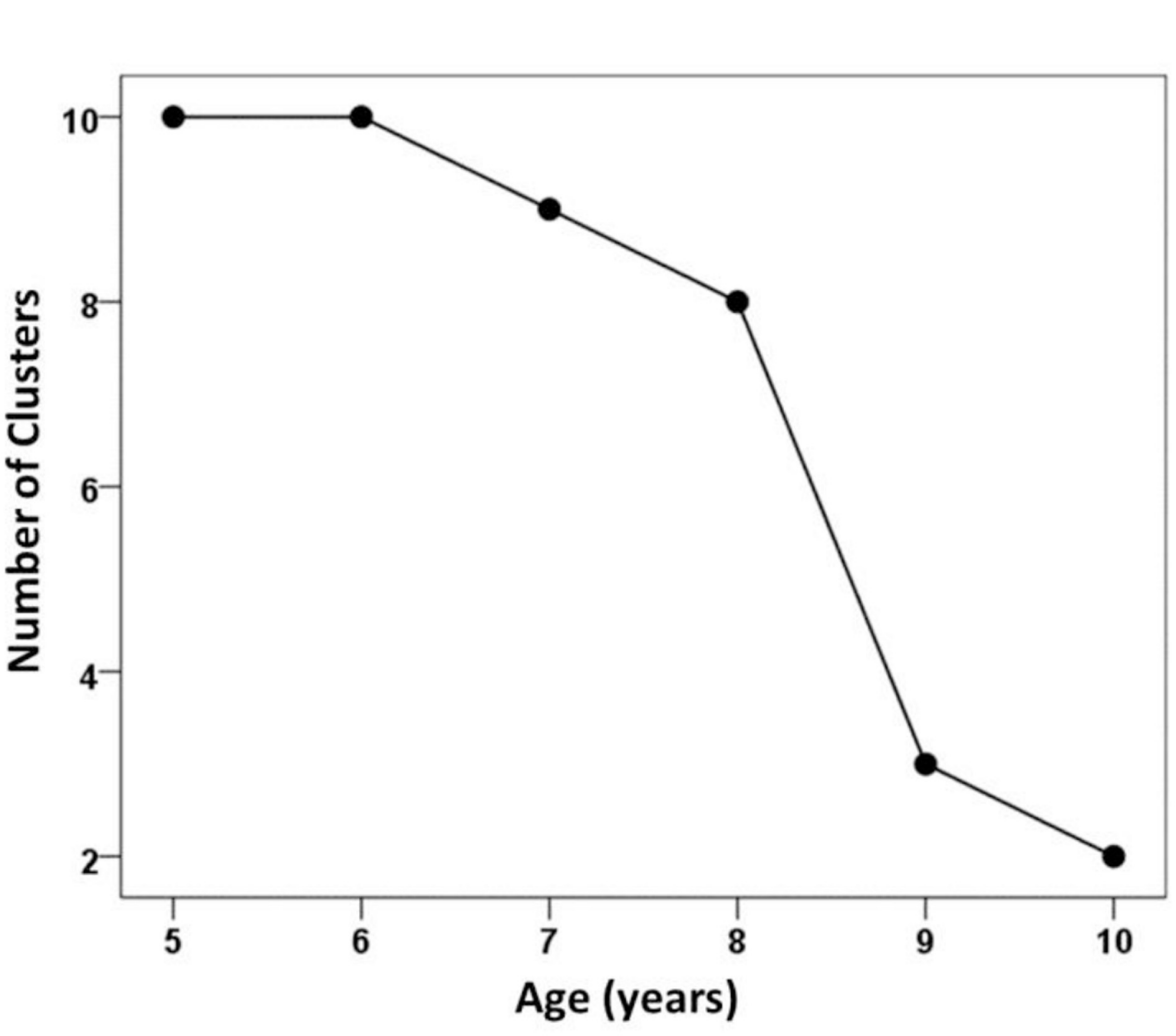
**The number of clusters plotted as a function of age**.

Figures 4, 5 illustrate the relationship between correct performance, variability, and chunking. As expected, overall performance improved with development, and became much less variable over the 6-year span (Figure 4). We also observed that the number of clusters decreased with age (Figure 5), thus demonstrating fewer chunks with development. Given the diversity of the tasks, this improvement can be viewed as the outcome of a process in which the children gradually became experts in spatial judgments across relational planes and verbal and nonverbal modalities. A number of organizing principles emerged in the tree structures which underlay chunking which can be used to characterize how development unfolds.

### Cognitive entropy

**Figure 6:**
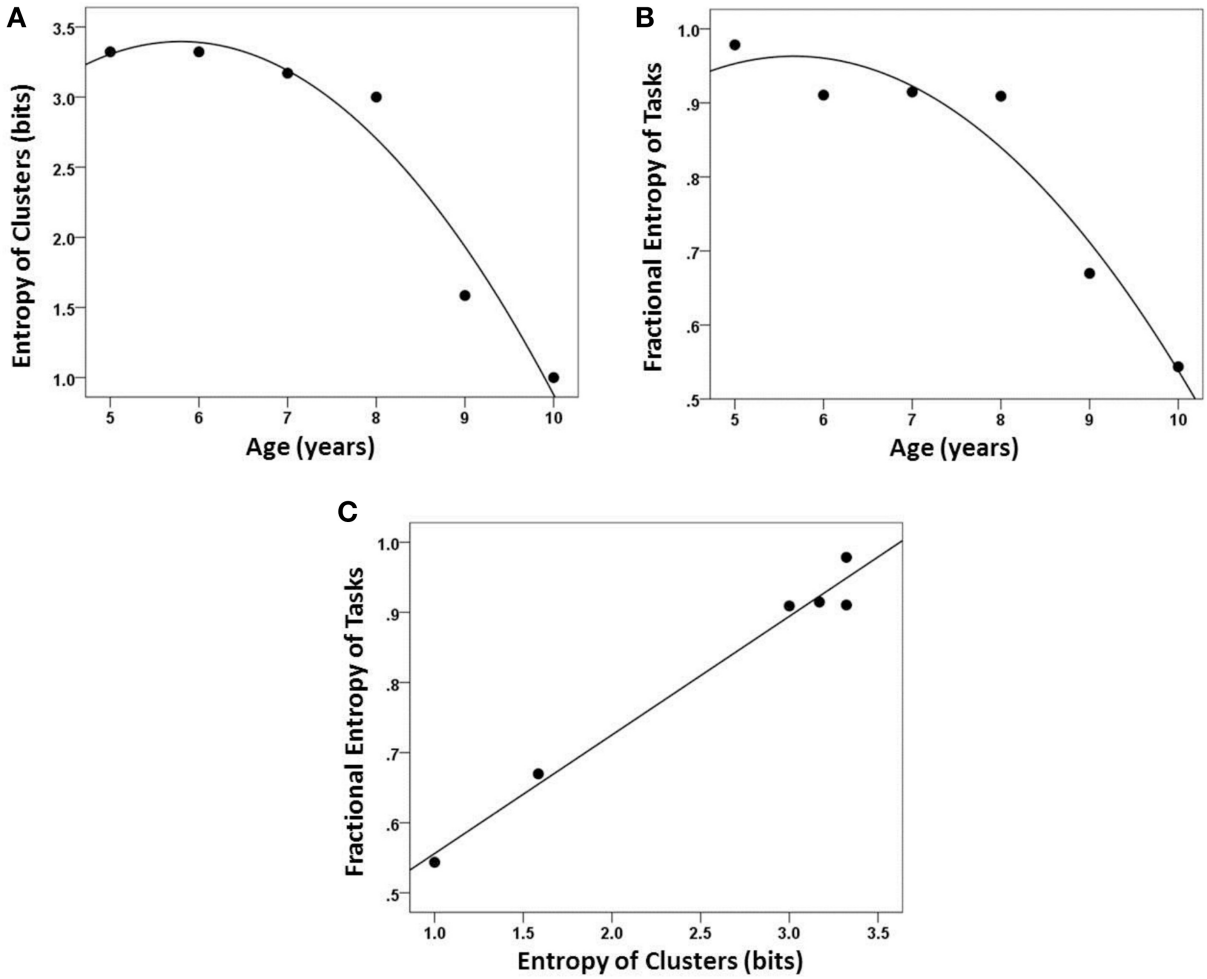
**Entropy measures. (A)** Entropy of chunking (*S*_*C*_) decreased with age. **(B)** Entropy of tasks across clusters (*S*′_*T*_) also decreased with age. **(C)** *S*_*C*_ and *S*′_*T*_ were highly correlated (*r* = 0.98).

**Figure 7:**
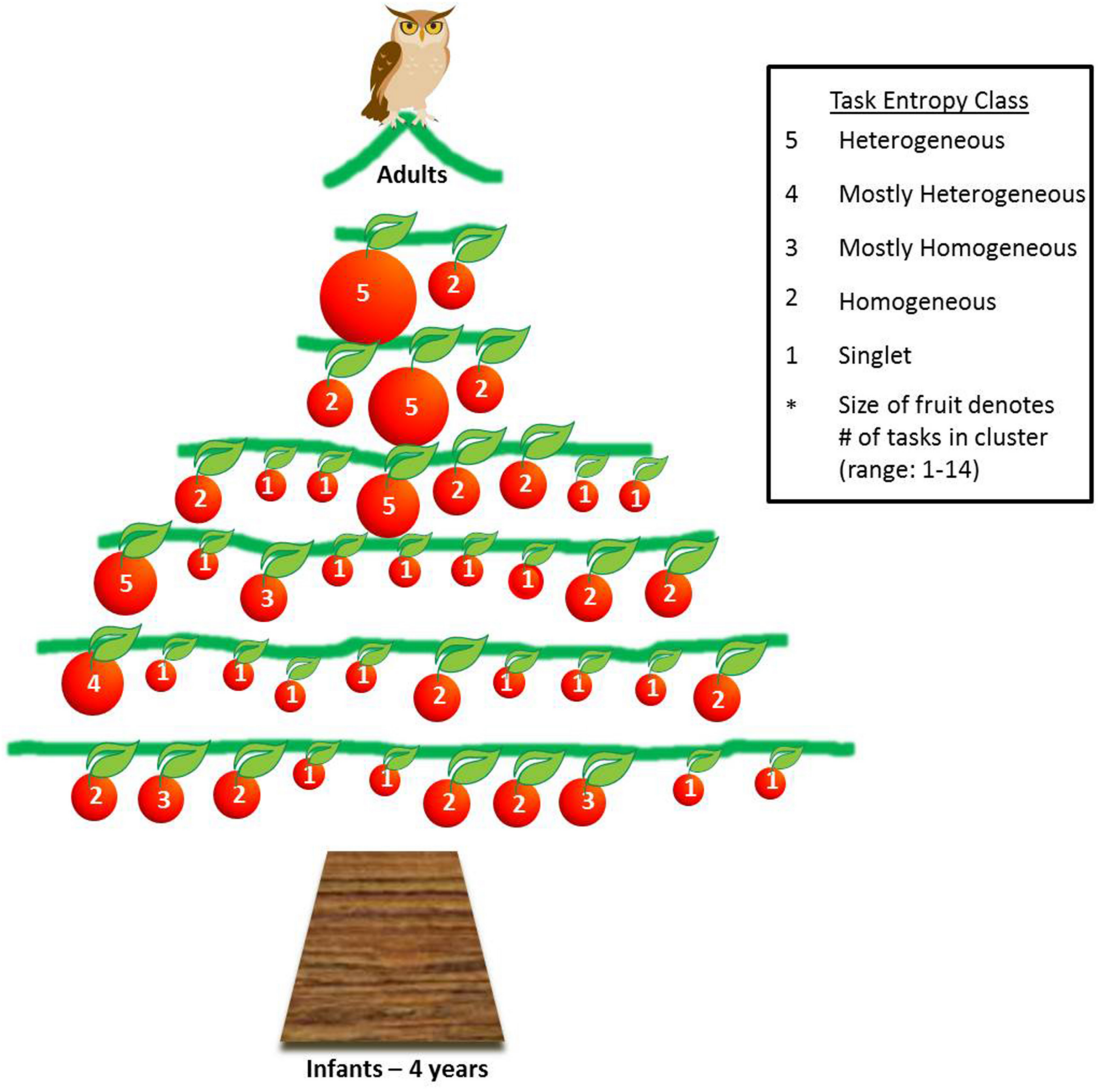
**Tree representing the development of spatial relational knowledge from 5 to 10 years of age**. Each branch represents one year of age, with 5 years as bottom branch and 10 years as topmost branch. Foundational spatial relational knowledge acquired between infancy and 4 years of age is represented as the trunk, although not investigated in this paper. The treetop and owl represent adults and the pinnacle of human spatial relational knowledge. Each fruit represents one cluster, the size of the fruit represents the number of items (tasks) within the cluster, and the quantity of fruit on each branch represents cluster entropy at each age. The number inside each fruit indicates the class of task entropy shown in the legend, which ranges from completely homogeneous (2) to completely heterogeneous (5). A cluster was defined as homogeneous if tasks within the cluster came from the same relational plane, same task condition and same task modality.

We quantified the incremental chunking process that emerged from our hierarchical clustering analyses using an information-theoretic framework where we measured the entropy in the cognitive organization of each age group. From the trees, we calculated both the cluster entropy within age group, *S*_*C*_, and the task entropy, *S*′_*T*_. Both *S*_*C*_ and *S*′_*T*_ decreased as a quadratic function of age (Figures 6A,B, respectively) and were positively, strongly and linearly related between themselves (Figure 6C). We provide an alternative graphical illustration of changing *S*_*C*_ and *S*′_*T*_ as a function of age in Figure 7, which may be helpful in visualizing the relationship between the two measures of entropy (especially in comparing ages 5 and 6, which have different distributions across the same number of clusters). These data also reveal an inflection point which suggests a relatively large cognitive gain between the ages of 8 and 9 years. Based on our findings, this is a transition to performing nonverbal tasks similarly to each other and similarly as well as *above/below* verbal tasks. In other words, the nonverbal tasks begin to merge with the verbal tasks.

**Figure 8:**
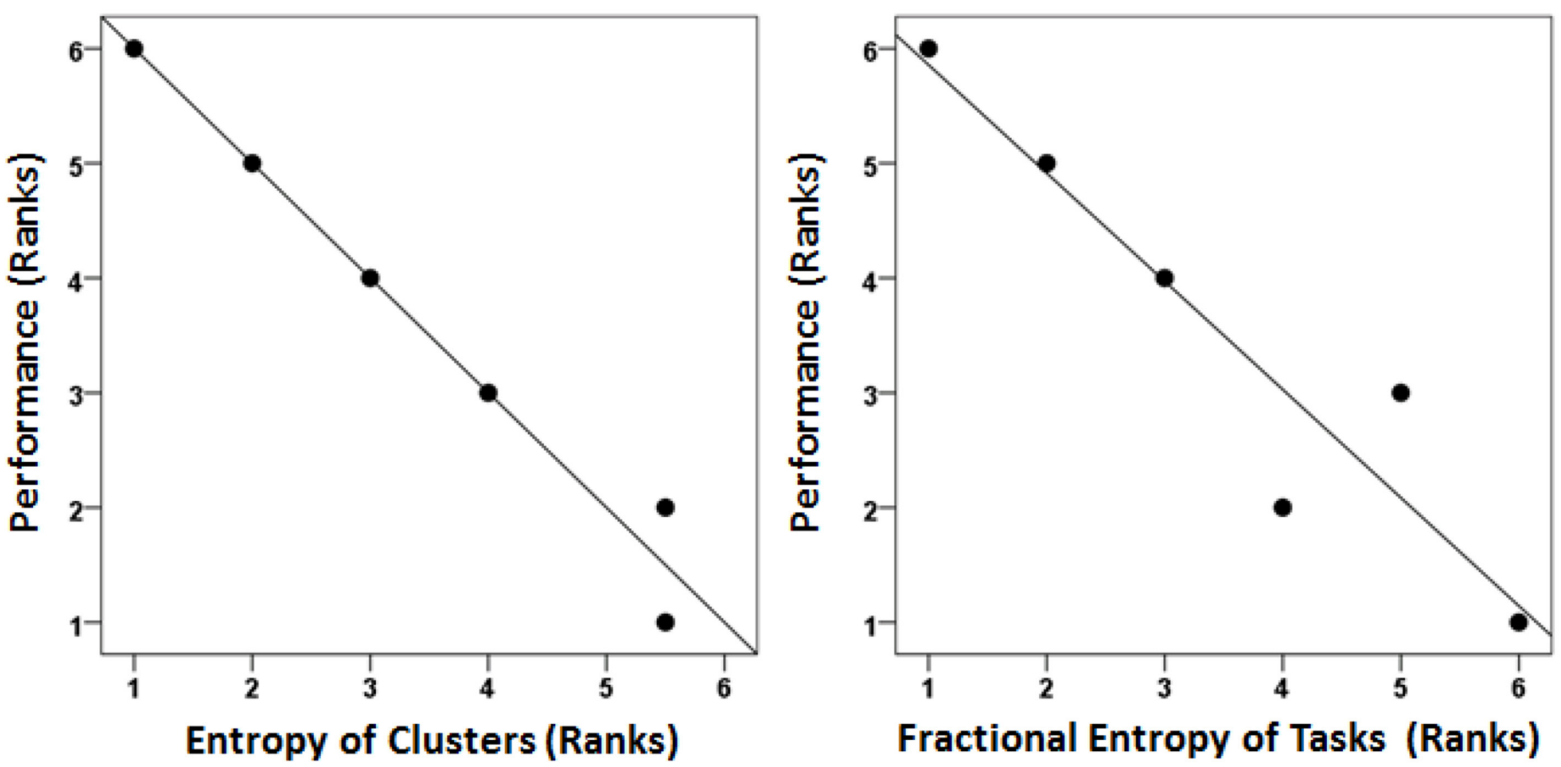
**Ranked correct performance was highly correlated with entropy reduction**. Each age group's correct performance was ranked. Its cluster and task entropies were also ranked. These ranks are and plotted against each other. (Spearman's rank correlation coefficient was 0.995 and 0.943 for the left and right panels, respectively).

We found an excellent correspondence between the ranked mean percent correct performance and both ranked *S*_*C*_ and *S*′_*T*_ (Figure 8, left and right panel, respectively). Although an overall better performance would be expected to be associated with a smaller number of clusters, i.e., a smaller *S*_*C*_ (since the clustering is based on variation in performance), the anisotropic distribution of tasks among clusters, reflected in *S*′_*T*_, should be independent of the overall performance level. However, ranked performance scores were highly correlated with both *S*_*C*_ and *S*′_*T*_ (Figure 8).

## Discussion

We believe that our analytic approach offers new insights into the process of cognitive development; namely, the mechanisms of restructuring (Piaget's assimilation and accommodation) that occur as knowledge is added to the cognitive system. In this paper, we have illustrated the application of this new approach using the development of relational knowledge in children. Our cluster analyses revealed the progressive treatment of different tasks as being similar (i.e., chunking), as these tasks became hierarchically organized with cognitive development. Our finding of entropy reduction captures the orderly gain of information on spatial relational judgments, as children gradually became experts in these judgments as indicated by their increasing performance accuracy. It should be remembered that the chunking measured in our cluster entropy cannot, by itself, dictate the outcome of our task entropy although the possible distributions of tasks across the chunks (clusters) is constrained by the number of available chunks. Taken together, our results also point to the operation of a basic “gain-of-knowledge” (i.e., reduction of entropy) process that drives both the chunking (*S*_*C*_ and the anisotropic distribution of tasks among the chunks (*S*′_*T*_), as evidenced by the high correlation between these two entropy measures. Such a process has been proposed previously on behavioral and theoretical grounds (Pascual-Leone, 1970; Piaget, 1977; Fischer, 1980). The orderly decrease of the number of clusters with age, their unequal sizes, and the diversity of their item membership all point to the chunking of information as key mechanisms of cognitive development. It is through consideration of which tasks chunked together at each age (i.e., stage of development) from which we can identify the task attributes on which the organizing principles act in organizing relational knowledge.

With this approach, we were able to capture the organization of relational knowledge as it was being built up from poorly connected (isolated pieces of) knowledge at age 5 years to nearly unified treatment of the different tasks at age 10 years, with an acceleration of chunking occurring between ages 8 and 9 years. Unlike previous studies (Miller, 1956; Ericsson et al., 1980; Feigenson and Halberda, 2008), our results addressed chunking of conceptual knowledge, not working memory. Previous work on memory has concentrated on children's (or adults') abilities to remember given information based on inherently chunking information into meaningful units that require fewer memory resources. For example, after 230 h of practice recalling a large list of numbers using mnemonic association (e.g., chunking number spans into dates, ages or running times), one man was able to increase his memory span from 7 to 79 items (Ericsson et al., 1980). To the best of our knowledge, our study is the first to apply this type of analysis to any area of conceptual development as a measure of cognitive organization.

Importantly, the diversity of our tasks, along multiple dimensions, provided the requisite variety for investigating how uncertainty in performing relational judgments becomes reduced with development, while the unevenness of children's learning of the basic spatial relational concepts allowed us to compare the development of these concepts across different modalities. If the tasks we chose were less differentiated in performance, especially at 5 years of age, then we would have found more chunks early in development and, thereby, would have less clarity in how concepts became organized and structured together. Crucially, our methods reveal the natural development of cognitive organization since children's strategies for each task were self-generated and under voluntary control rather than relying on the strategy provided by an adult (e.g., children were not given labels prior to any task, except the verbal comprehension task).

Through our illustrated application, we showed how cluster analyses could be used to extract empirically significant information from task performance. Specifically, we used children's performance on 16 tasks to cluster performances and then assessed the clusters to discover under what principles performances were being organized and chunked. Our findings indicated that once a chunk was formed it was robust, meaning that it was likely to appear at each subsequent age and that the chunked items continued to be strongly associated. Therefore, once two tasks were performed similarly within one age group, all subsequent ages continued to show strongly similar performance on those two tasks. This was especially true for the verbal tasks. Furthermore, previous research has shown that a chunk can contain any number of concepts that share strong associations to one another (reviewed in Cowan, 2001). This means that a chunk need not form from purely similar concepts (e.g., only verbal tasks or only instances of *above*), but it does suggest that once two items are chunked together then those items should continue to be strongly associated and, therefore, chunked. Additionally, the robustness of specific clusters (i.e., content) across the ages (e.g., production of *above/below*) as revealed by the cluster analyses adds qualitative support to our quantitative findings from the entropy measures (see cluster content in Figure 3). This all points to irreversibility as an important aspect of cognitive organization; that once items group, they rarely ungroup.

Importantly, this method provides new insights into knowledge development by revealing the step-by-step progression of organization across age and illustrating how development progresses in an order that does not appear to be logical. To illustrate, the tasks in this study could have chunked according to any of 4 attributes—namely, modality (verbal/nonverbal), task condition (verbal production/comprehension, nonverbal congruent/incongruent), relational plane (*above/below, right/left*) or relation (*above*, *below*, *left*, *right*). It could be logically assumed that chunks would first form by relation if the concept of *above* is consistent across task conditions and modalities. However, our evidence suggests concepts first got chunked by task condition and relational plane (where opposites formed a single chunk), and later across task conditions but within task modality. For example, chunks at early ages (i.e., 5–7 years) were primarily within task and within plane, and at later ages tasks began to chunk within modality (i.e., nonverbal congruent and incongruent tasks began to merge at 8 years). Our finding that the relational planes were important organizing principles for chunking the verbal tasks, but not for chunking the nonverbal tasks, suggests that the division between the horizontal and vertical planes exists only linguistically, and not nonverbally. Consistent with our findings, previous research has suggested that children know that two labels are spatial opposites, before they can accurately marry the labels to the concepts (i.e., label specific poles, Clark, 1972). More broadly, our findings suggest that the verbal and nonverbal instantiations of a concept do not follow the same organizing principles, nor does it appear that the *above/below* and *right/left* relational planes follow similar organizing principles as each other. Our findings may suggest that the label plays a role in restructuring the concept and assimilate them to fit with the label—at least this seems to be true for *above* and *below*.

Producing the terms *right* and *left* remained separate from all other tasks, even for 10-year-olds. This suggests that production of *right* and *left* fails to generalize into a common factor with the other tasks and with the production of the terms *above* and *below*. This may be why adults continue to confuse *right* and *left* (Wolf, 1973; Hannay et al., 1990) at times. It is unclear at what age the production of *right* and *left* become chunked with the other tasks or whether these terms ever fully merge with their corresponding nonverbal concepts. The current decision-making models that have been developed to explain spatial relational judgments at the neurobehavioral level (Regier and Carlson, 2001; Lipinski et al., 2012) do not account for this differential performance across the two planes.

Globally, our approach offers a way of operationalizing and quantifying developmental processes across all domains of knowledge—spatial, perceptual, linguistic, conceptual and social domains—with broad, practical applications. Examples of broad applications include discovering which concepts are needed for other concepts to be gained or the order in which different elements of cognition (e.g., working memory, self-recognition, language, cognitive control, etc.) come online, while more specific applications include finding how labels are attached to concepts. In short, our methods could reveal additional information on how cognitive abilities are developing and getting honed. By looking at the gradual progression of knowledge and skill learning, results from this approach can unravel developmental processes which can in turn be used to alter educational models to coincide more naturally to how children are actually acquiring skills and knowledge. This approach can be used to uncover the foundation for all knowledge and this approach should be applied within other cognitive domains and across domains.

Specifically, our findings for spatial relational knowledge have immediate, direct application in education. For example, knowing how relational skills are built informs academic curricula development. Current research suggests that spatial skills are often overlooked in curricula creation and implementation, although training in spatial thinking can improve achievement in certain disciplines, such as mathematics and sciences (Uttal et al., 2013b; Stieff et al., 2014). Specifically, our findings suggest that such training might begin by verbally pairing up polar opposites (e.g., *above*/*below, right/left*), in concordance with previous findings that suggest children learn two words are opposites before they fully map the terms to their relations (Clark, 1972). Only after opposites are mastered children might be taught to make nonverbal perceptual matching judgments, like our nonverbal congruent trials. Finally, these congruent judgments should be the basis for teaching how to make the more difficult incongruent relational judgments. In short, our findings suggest how a unified system of knowledge for making relational judgments might be built—by appealing to the natural progression by which these skills develop.

## Author contributions

All authors developed the study concept and contributed to the study design. Testing and data collection were performed by Nicole M. Scott. Apostolos P. Georgopoulos and Nicole M. Scott performed the data analysis and interpretation. Nicole M. Scott drafted the manuscript, and Apostolos P. Georgopoulos and Maria D. Sera provided critical revisions. All authors approved the final version of the manuscript.

### Conflict of interest statement

The authors declare that the research was conducted in the absence of any commercial or financial relationships that could be construed as a potential conflict of interest.
